# A Convalescent Home on New Lines

**Published:** 1901-08-17

**Authors:** 


### A CONVALESCENT HOME ON NEW LINES.

A convalescent home that will not be dependent on charity for its continued existence is an interesting experiment. One such has just been opened at Roden, near Shrewsbury, on an estate of 740 acres purchased five years ago by the directors of the English Co-operative Wholesale Society, and turned into a fruit farm. Included in the purchase was a fine residential hall, formerly let with shooting rights, and when the directors came before the shareholders with a proposal to convert the hall into a convalescent home, they unanimously approved, and the sum of £10,000 was readily granted for the purpose of carrying out the undertaking. New wings have been added, and at present twenty-five visitors can be received. The bedrooms are lofty and airy, the sanitary arrangements are good, the electric light is installed throughout, and there is a fireproof staircase at the back of the building. Among other luxuries there is a drawing-room for ladies, and recreation and smoking rooms for men; the latter a glass-covered apartment leading into the grounds. There is also a billiard-room, and the dining-room will accommodate fifty persons. The furnishing has been carried out under the supervision of Mr. Allen, of the Furnishing Department of the Manchester Stores. A modern steam laundry adjoins the building, which is surrounded by gardens and lawns, and from which a distant view of the Wrekin may be obtained. There is also a kitchen-garden, and a well-filled vinery.

At the opening ceremony on the 27th ult. Mr. Shillito, J.P. (chairman), said there was an idea among business men that those engaged in commerce could not enter into philanthropy without jeopardising their influence as traders, but he did not think that what they were inaugurating would in any way minimise their influence as a large trading body. Co-operators were democratic, and they looked upon kindness and benevolence to their kith and kin as part of their duty. When the directors purchased the Roden estate they saw at once that it had one distinct quality—it was highly climatic. They had met with a ready response from the shareholders when they proposed to convert the hall into a convalescent home. The water supply had been improved, and the original price of the estate had been considerably increased by the building of cottages for labourers, the erection of greenhouses, etc. They did not expect to make a dividend, but they were demonstrating in a small way what the working classes, when organised, could do with the land. They had every confidence that the home would be a success, and trusted that that day would mark the dawn of a new era in the development of the benevolent and humanising side of co-operation.

Mr. G. Hawkins, in proposing "The Success of the Home," said it was certain that the opening would have a very far-reaching effect on the operations of the wholesale and distributive societies, whose members, when broken down, would find their way to Roden. It would, he thought, lift the movement out of the rut of mere dividend-making. The directors of the Wholesale Society ought to be proud to have set the pace in regard to the establishment of these homes in England.

The building operations have been carried out under the direction of Mr. Harris, the society's architect. The terms of admission are that "the intending visitor must be a *bonâ-fide* convalescent and a member of a society affiliated with the Wholesale Society." The matron is Miss Twigg.

